# Publisher Correction: Expression and regulation of SETBP1 in the song system of male zebra finches (*Taeniopygia guttata*) during singing

**DOI:** 10.1038/s41598-025-89141-7

**Published:** 2025-02-12

**Authors:** Dana Jenny Grönberg, Sara Luisa Pinto de Carvalho, Nikola Dernerova, Phillip Norton, Maggie Mei-Ki Wong, Ezequiel Mendoza

**Affiliations:** 1 Institut für Verhaltensbiologie, Freie Universität Berlin, 14195 Berlin, Germany; 2 Institute for Theoretical Biology, Humboldt-Universität zu Berlin, Philippstr. 13, Haus 4 (Ostertaghaus), 10115 Berlin, Germany; 3 Language and Genetics Department, Max Planck Institute for Psycholinguistics, Nijmegen, 6500AH the Netherlands

Correction to: *Scientific Reports* 10.1038/s41598-024-75353-w, published online 23 November 2024

In the original version of this Article Maggie Mei-Ki Wong and Ezequiel Mendoza were omitted as equally contributing authors.

The original Article has been corrected.

