# Supplementary material for: Expression and regulation of SETBP1 in the song system of male zebra finches (Taeniopygia guttata) during singing
Source: Sci Rep. 2024 Nov 23;14:29057. doi: 10.1038/s41598-024-75353-w (PMC11585544; doi:10.1038/s41598-024-75353-w)
Supplement: Supplementary file 1 — Supplementary Material 1 [file 41598_2024_75353_MOESM1_ESM.docx]

Title: **Expression and regulation of SETBP1 in the song system of male zebra finches (*Taeniopygia guttata*) during singing**

Abbreviated title: SETBP1 in male zebra finches

Author names and affiliations, including postal codes

Dana Jenny Grönberg ^1^ <https://orcid.org/0009-0004-3231-778X>; Sara Luisa Pinto de Carvalho ^1^ <https://orcid.org/0009-0005-8457-1760>; Nikola Dernerova ^1^ <https://orcid.org/0009-0006-3589-5782>; Phillip Norton ^2^ <https://orcid.org/0000-0002-3137-2582>; Maggie Mei-Ki Wong ^3, #^ <https://orcid.org/0000-0002-9438-0141> and * Ezequiel Mendoza ^1, #^ <https://orcid.org/0000-0003-4963-519X> .

^1^ Institut für Verhaltensbiologie, Freie Universität Berlin, 14195 Berlin, Germany

^2^Humboldt-Universität zu Berlin, Institute for Theoretical Biology, Philippstr. 13, Haus 4 (Ostertaghaus), 10115 Berlin, Germany

^3^ Language and Genetics Department, Max Planck Institute for Psycholinguistics, 6500AH Nijmegen, the Netherlands

# Contribute equally

Corresponding author email address: * Dr. Ezequiel Mendoza emendoza@zedat.fu-berlin.de

**Supplementary table 1**. Amino acid comparison of zebra finch and human SETBP1 protein domains

| **Domain** | **aa (IsoA)** | **aa human protein (Morgan et al 2021)** | **% similarity** |
| --- | --- | --- | --- |
| AT-Hook domain 1 | 625-637 | 584–596 | 100 |
| AT-Hook domain 2 | 1058-1070 | 1016–1028 | 100 |
| AT-Hook domain 3 | 1490-1502 | 1451–1463 | 85 |
| HCF1 binding motiv | 1033-1036 | 991–994 | 100 |
| NLS 1 | 505-520 | 462–477 | 56 |
| NLS 2 | 1409-1423 | 1370–1384 | 53 |
| NLS 3 | 1422-1438 | 1383– 1399 | 65 |
| SET-binding domain | 1332-1527 | 1292–1488 | 80 |
| SKI homologous region | 748-959 | 706– 917 | 92 |
| PEST 1 | 1-13 | 1-13 | 92 |
| PEST 2 | 307-318 | 269–280 | 50 |
| PEST 3 | 589-601 | 548– 561 | 69 |
| PEST 4 | 719-731 | 678–689 | 92 |
| PEST 5 | 848-872 | 806–830 | 96 |
| PEST 6 | 1541-1565 | 1502–1526 | 92 |
| PPLPPPPP 1 | 1563-1570 | 1520-1527 | 100 |
| PPLPPPPP 2 | not present | 1528-1535 | / |
| PPLPPPPP 3 | not present | 1536-1543 | / |

**
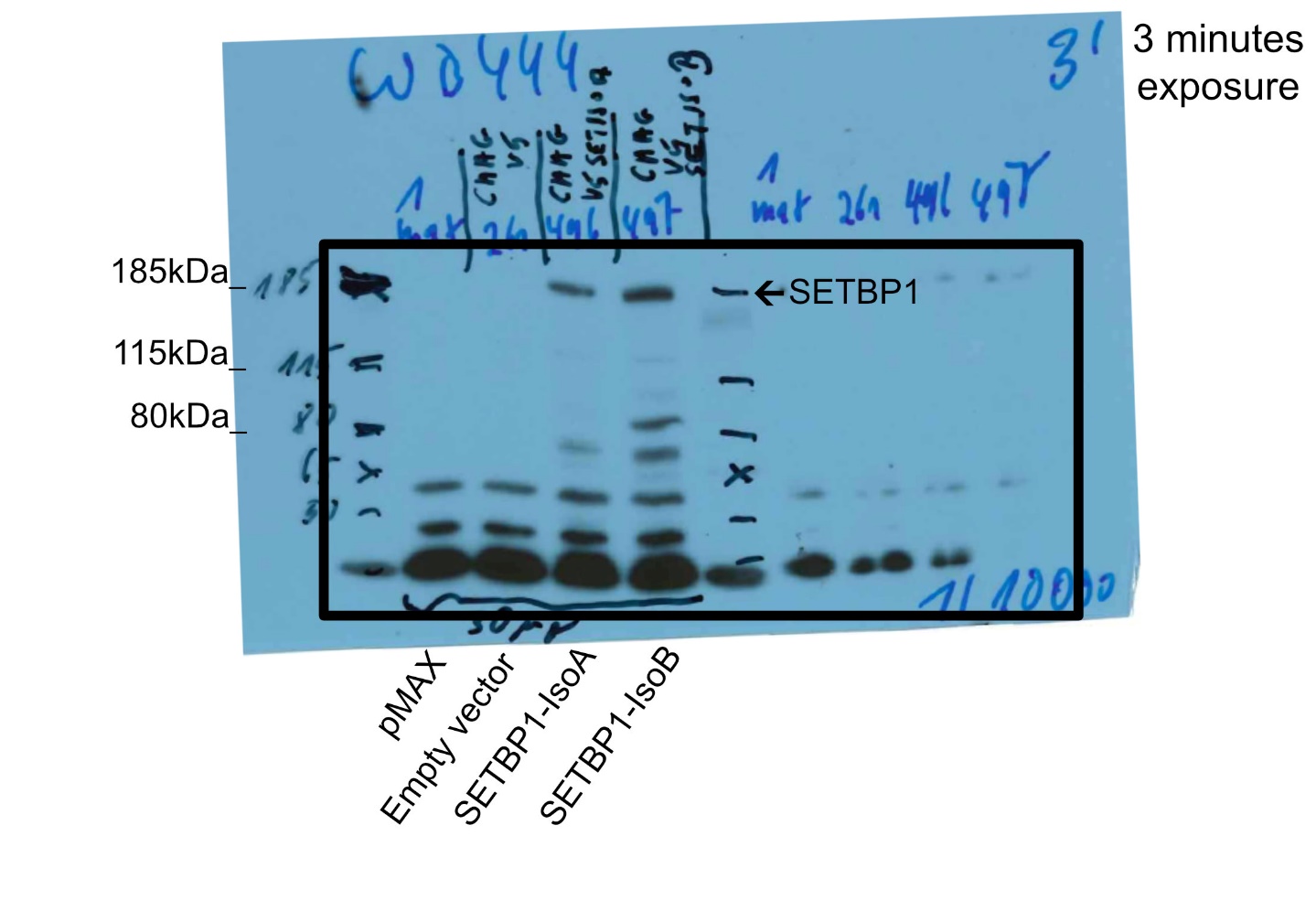
Supplementary figure 1.-** Original Western blot detection film from which the cropped Figure 2a was taken. The left half of the gel, up to the marker in the middle, was loaded with 30 µg protein lysate and is the portion shown in Figure 2a. The right half of the gel was loaded with 10 µg protein lysate, but the bands of SETBP1 could not be detected and therefore this half was not chosen. The loading order on both sides is the same, starting from left to right: marker, pMAX, empty vector, zebra finch SETBP1-isoA, and zebra finch SETBP1-isoB, all detected with the SETBP1 antibody at a concentration of 1:10,000. The exposure time of this film was 3 minutes. Since the borders of the gel are not visible, we have indicated the borders with a black line.


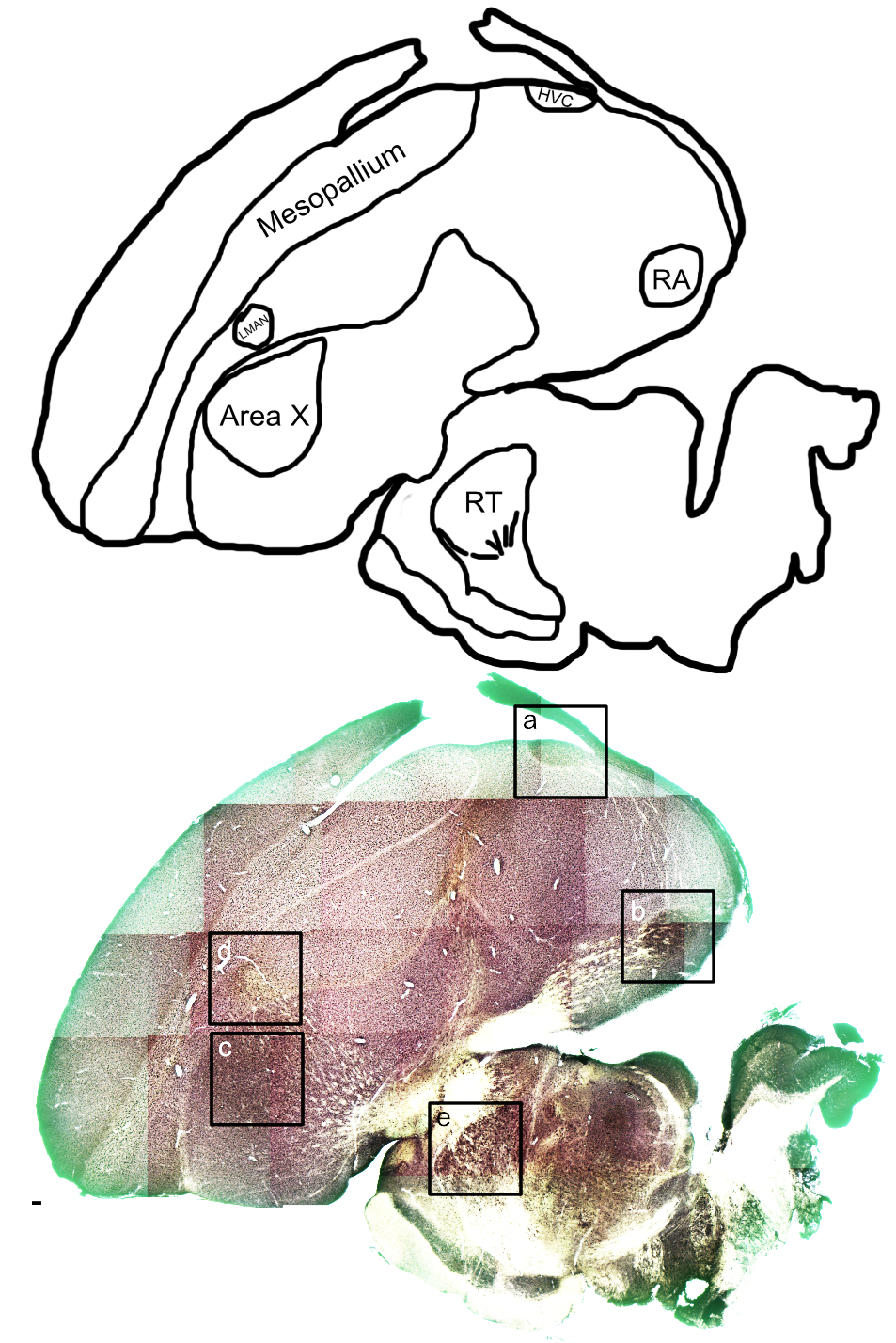


**Supplementary figure 2.-** Expression pattern of zebra finch SETBP1 protein after DAB immunodetection in a non-singing adult male (related to Figure 4). Upper part shows a schematic representation of the brain slice pointing out the regions where higher magnification photos were taken or are mentioned in the article. Names and locations of song system nuclei are shown: HVC (4a), nucleus robustus arcopallii (RA, 4b), Area X (4c), nucleus lateralis magnocellularis nidopallii anterioris (LMAN, 4d), and nucleus rotundus (RT, 4e), as well a mesopallium the region where figure 3 photos were taken. Lower part is a representative brightfield photomicrographs of a sagittal section detected with an antibody against SETBP1 and revealed with nickel enhanced DAB staining. HVC, RA, and Area X show a darker staining than the surroundings, LMAN has a weaker staining than surrounding. Higher magnification photos of figure 4 were taken from this slice (squares show the regions where the higher magnification photos were taken) and an adjacent slice was the NPC also shown in Figure 4. Scale bar = 100µm.


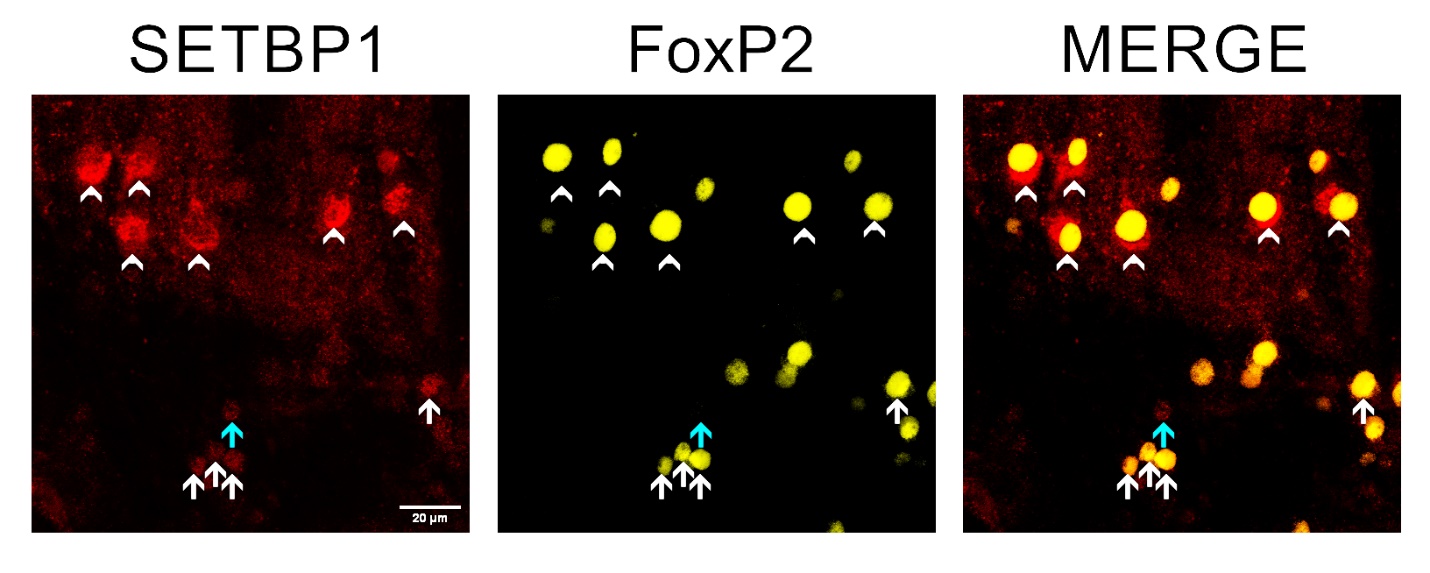


**Supplementary figure 3.-** Fluorescent immunodetection of zebra finch SETBP1 showing nuclear and cytoplasmic expression in the region of nucleus rotundus (RT) and co-localization with FoxP2. Zebra finch SETBP1 (color-coded in red) and FoxP2 (pseudo- color-coded in yellow) immunostainings showing co-localization examples in RT. Zebra finch SETBP1 is mostly expressed cytoplasmic in RT region (arrowheads) and co-localize with FoxP2, in contrast to the surrounding tissue where zebra finch SETBP1 is nuclear and may be co-localized with FoxP2 (white arrows) or not (blue arrows). Scale bar = 20µm.

**Supplementary table 2**.- ANOVA and Tukey post hoc *p*values of co-localization of SETBP1 with either FoxP1, or FoxP2 or Pv (related to Figure 6).

|  | **FoxP1 / SETBP1** | **FoxP2 / SETBP1** | **Pv / SETBP1** |
| --- | --- | --- | --- |
| **ANOVA *p*-value** | 2.339 | 0.5604 | 0.2980 |
| **Treatment pair** | **Tukey *p*-value** | **Tukey *p*-value** | **Tukey *p*-value** |
| **DS vs US** | 0.8999947 | 0.8999947 | 0.8999947 |
| **DS vs NS** | 0.2684999 | 0.7210704 | 0.8863397 |
| **DS vs Juv** | 0.8999947 | 0.6647150 | 0.3041684 |
| **US vs NS** | 0.2369559 | 0.7730901 | 0.8999947 |
| **US vs Juv** | 0.8999947 | 0.7085287 | 0.3399811 |
| **NS vs Juv** | 0.3973985 | 0.8999947 | 0.7299706 |
